# Thromboelastography Maximum Amplitude Is a Valuable Biomarker for Early Atherosclerosis in Rheumatoid Arthritis Patients: A Single‐Center Cross‐Sectional Study

**DOI:** 10.1002/kjm2.70043

**Published:** 2025-05-08

**Authors:** Qing‐Lin Zhang, Qian Xu, Rong Huang, Ming‐Zhong Sun, Dong‐Mei Jiang, Hui Tao, Hao Jin

**Affiliations:** ^1^ Department of Blood Transfusion Yancheng Third People's Hospital, the Affiliated Hospital of Jiangsu Vocational College of Medicine, Affiliated Hospital 6 of Nantong University Yancheng Jiangsu China; ^2^ Department of Clinical Laboratory Yancheng Third People's Hospital, the Affiliated Hospital of Jiangsu Vocational College of Medicine, Affiliated Hospital 6 of Nantong University Yancheng Jiangsu China; ^3^ Department of Endocrinology Yancheng Third People's Hospital, the Affiliated Hospital of Jiangsu Vocational College of Medicine, Affiliated Hospital 6 of Nantong University Yancheng Jiangsu China

**Keywords:** atherosclerosis, maximum amplitude, rheumatoid arthritis, thromboelastography

## Abstract

Hypercoagulability contributes to atherosclerosis (AS) progression in individuals suffering from rheumatoid arthritis (RA), and thromboelastography (TEG) is a valuable diagnostic tool. This hospital‐based cross‐sectional study assessed the clinical efficacy of TEG parameters in detecting hypercoagulability and subclinical AS by purposively sampling 372 RA patients and 105 healthy controls. TEG indices, conventional coagulation markers, and biochemical profiles were assessed. Multivariable logistic modeling and receiver operating characteristic (ROC) analyses were utilized to identify independent risk factors for early AS in patients with RA. Our results indicated that patients with RA experienced a hypercoagulable state in comparison to controls, which was particularly pronounced in RA patients with early AS compared to their non‐AS counterparts. Logistic regression analysis revealed that the TEG maximum amplitude (MA) is an independent risk factor for early AS in patients with RA, with an odds ratio of 1.219 and a 95% confidence interval of 1.128–1.317. Additional independent risk factors identified are fibrinogen (FIB), low‐density lipoprotein cholesterol (LDL‐C), and the adjusted atherogenic index of plasma (aAIP). ROC analysis revealed that MA achieved an area under the curve (AUC) of 0.711 (sensitivity: 71.5%; specificity: 63.5%) for early AS diagnosis. Combining with FIB, LDL‐C, and aAIP significantly enhanced diagnostic performance, with an AUC of 0.831 (sensitivity: 77.6%; specificity: 75.3%). These findings highlight MA as a valuable biomarker for early detection of AS in patients with RA. Furthermore, integrating MA with FIB, LDL‐C, and aAIP improves diagnostic accuracy, supporting its clinical role in risk stratification and early intervention.

## 1 Introduction

Rheumatoid arthritis (RA) is a long‐term autoimmune disorder marked by ongoing inflammation of the synovium, resulting in progressive joint damage [1]. Besides joint manifestations, RA is marked by widespread inflammation, impaired endothelial function, and lipid metabolism dysregulation, all of which contribute to a hypercoagulable state that can precipitate thrombosis and/or cardiovascular disease (CVD) [2, 3, 4]. Consequently, patients with RA frequently exhibit varying degrees of coagulation and fibrinolysis abnormalities.

Atherosclerosis (AS) constitutes the fundamental pathological underpinning of CVD, with its incipient stages identifiable through the assessment of carotid intima‐media thickness (CIMT). This measurement has been proposed for cardiovascular risk stratification across various chronic conditions, including RA [5, 6]. AS pathogenesis and progression are influenced by many factors, including chronic inflammation, macrophage activation, lipid accumulation, endothelial dysfunction, and coagulation disorders [7, 8, 9]. Consequently, a hypercoagulable state, precipitated by an impaired coagulation system, may interact synergistically with inflammatory processes and other pathophysiological mechanisms, facilitating the progression of AS, which may lead to fatal and nonfatal cardiovascular events in patients with RA. Presently, thromboelastography (TEG) is recognized as a validated and reliable technique for assessing hypercoagulability [10]. TEG provides a comprehensive assessment of coagulation and offers a more dynamic evaluation of coagulation function than conventional coagulation tests [11]. TEG can measure hypo‐ or hypercoagulability parameters, including kinetics (K) time, reaction (R) time, maximum amplitude (MA), and α angle. Hypercoagulable or prothrombotic states are typically characterized by increased MA and α angle, along with shortened R time and K time [12, 13]. In conventional coagulation tests, hypercoagulability may be indicated as shortened prothrombin time (PT), activated partial thromboplastin time (APTT), and thrombin time (TT), as well as increased D‐dimer and fibrinogen (FIB) levels [14, 15].

Despite the implication of a hypercoagulable state in AS development and progression, a thorough study of the association between coagulation parameters, including TEG and AS, in individuals with RA has not yet been undertaken. This study aimed to identify relevant blood coagulation parameters in early AS in patients with RA and assess their role in the differential diagnosis of early AS.

## 2 Materials and Methods

### 2.1 Ethics Statement

This study obtained approval from the Medical Ethics Committee of Yancheng Third People's Hospital (No. 2021‐030‐01). Informed consent was obtained from all participants prior to this study.

### 2.2 Study Population

**FIGURE 1:**
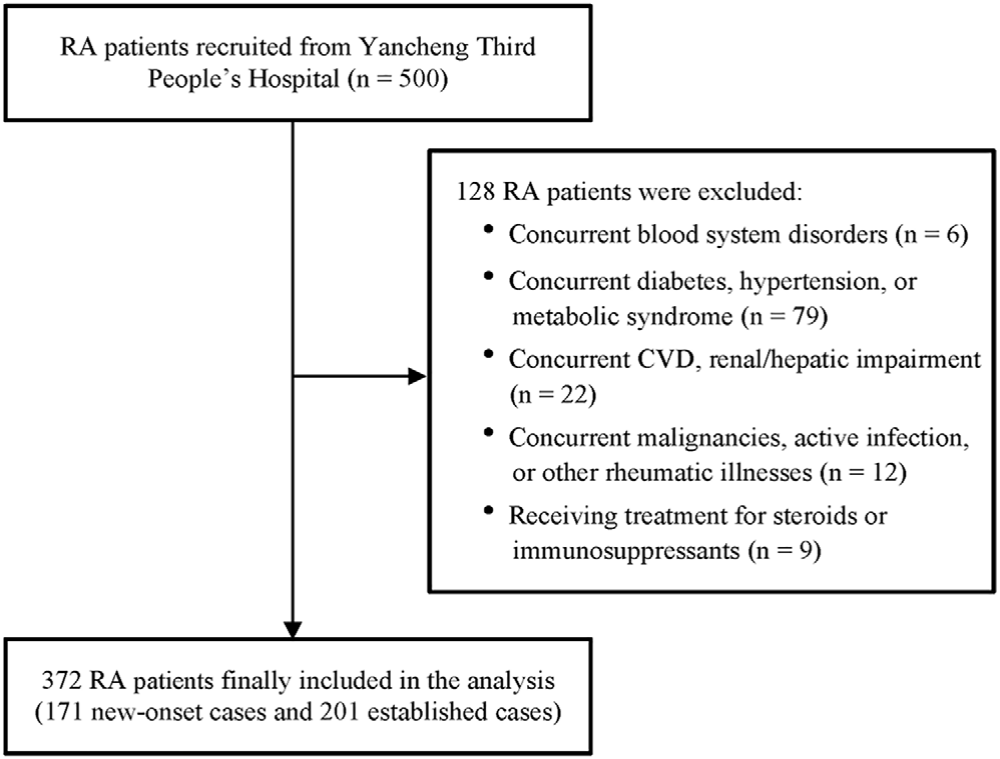
The flowchart of patient inclusion and exclusion.

We recruited 500 patients with RA admitted to Yancheng Third People's Hospital between July 2019 and June 2023. All patients met the 2010 American College of Rheumatology (ACR) diagnostic criteria for RA [16]. Following rigorous screening, 128 participants were excluded based on predefined criteria: 6 with concurrent blood system disorders, 79 with comorbidities including diabetes, hypertension, or metabolic syndrome, 22 with CVD or renal/hepatic impairment, 12 with malignancies, active infections, or other rheumatic conditions, and 9 receiving steroid or immunosuppressive therapy within the last 3 months. Ultimately, this study included 372 patients with RA: 171 new‐onset cases (disease duration < 6 months) and 201 established cases. Figure 1 illustrates a detailed flowchart of the entire patient selection process. Additionally, 105 healthy controls without RA diagnosis, comorbidities, or relevant medication history were included in the comparative analysis.

### 2.3 Major Instruments and Reagents

The primary instruments and reagents used in this study were as follows: the biochemical immunoassay system (cobas 8000 modular analyzer series, Roche Diagnostics, Switzerland) with its reagents, calibrators, and quality control products; a thromboelastogram analyzer (Chongqing DingRun, Chongqing, China) equipped with proprietary coagulation reagent cartridges and quality control materials; an automated coagulation analyzer (CS‐5100, Sysmex, Japan) and its supporting reagents; and a high‐resolution B‐mode carotid ultrasound imaging system (Philips EPIQ5, Philips Healthcare, USA).

### 2.4 Data Collection

Several anthropometric variables were measured by trained staff, encompassing stature, body weight, blood pressure, and body mass index (BMI). The formula for BMI is body weight in kilograms divided by the stature in meters squared. An analysis of fasting blood samples was performed by the fully trained laboratory technicians to determine the concentrations of fasting blood glucose (FBG), high‐density lipoprotein cholesterol (HDL‐C), low‐density lipoprotein cholesterol (LDL‐C), triglycerides (TG), total cholesterol (TC), and high‐sensitivity C‐reactive protein (hs‐CRP). To calculate the atherogenic index of plasma (AIP), we used the formula: AIP = log (TG/HDL‐C). To avoid negative AIP values, the adjusted AIP (aAIP) was calculated by first multiplying TG/HDL‐C by 10, and then taking the logarithm, that is, aAIP = log (TG/HDL‐C*10) [17].

Technicians with professional training performed TEG and conventional coagulation assays. The conventional coagulation assays measured the following parameters: PT, APTT, TT, D‐dimer, and FIB. Standardized TEG procedures were adhered to in accordance with the manufacturer's guidelines, and test results were obtained within 2 h of blood sample collection. This study assessed the following TEG parameters: R time, K time, α angle, and MA. The significance of each parameter is as follows: R time (5–10 min) indicates the duration required for clot initiation, while K time (1–3 min) indicates the rate of clot formation during the R time until a clot firmness of 20 mm is achieved. The α angle (53°–72°) represents the tangent of the curve, indicating the clot formation rate. MA (50–70 mm) indicates the strength of the developing clot, primarily influenced by platelet function and count [12, 13].

CIMT and carotid plaque presence were assessed by skilled ultrasonographers. During the procedure, patients were positioned supine with gentle neck rotation to allow for sequential examination of the main carotid vessels: the common, external, and internal carotid arteries, as well as the carotid bifurcation area. CIMT measurements were obtained for the arterial walls. Measurements were taken from the two sides to assess early AS. According to existing literature, CIMT values > 0.9 mm indicate thickening [18].

### 2.5 Statistical Analysis

The data were analyzed using SPSS software (Version 27.0). The Kolmogorov–Smirnov test was first applied to check whether continuous variables adhere to a normal distribution. For variables that follow a normal distribution, their characteristics are typically summarized using the mean and standard deviation (SD). On the other hand, when variables exhibit a non‐normal distribution, they are described using the median, along with the interquartile range. To compare these variables, data that follow a normal distribution are analyzed using unpaired *t*‐tests, while data that do not follow a normal distribution are analyzed using Mann–Whitney *U* tests. Chi‐square tests were performed for categorical variables. The independent risk factors for early AS in RA patients were identified by performing a multivariate analysis using a logistic regression model, with CIMT > 0.9 as the dependent variable and demographic and clinical parameters that demonstrated statistical significance in the univariate analysis as independent variables. The independent variables' multicollinearity was evaluated with the help of the variance inflation factor (VIF), excluding those with a VIF ≥ 5. To assess the diagnostic value of the associated parameters for early AS, receiver‐operating characteristic (ROC) curve analyses were performed to determine the area under the curve (AUC), the optimal cut‐off value, sensitivity, and specificity. A two‐tailed *p* value that is below 0.05 is regarded as statistically significant.

## 3 Results

### 3.1 Comparison of Characteristics Between RA Patients and Healthy Controls

The demographic, anthropometric, and laboratory data of 392 patients with RA and 105 healthy controls are summarized in Table 1. The differences between patients and controls regarding age, gender, systolic blood pressure (SBP), diastolic blood pressure (DBP), FBG, HDL‐C, PT, TT, R time, K time, and α angle were statistically non‐significant (*p* > 0.05). Patients with RA exhibited significantly higher mean MA (62.31 ± 4.60 mm vs. 61.20 ± 4.45 mm) and CIMT values (0.94 ± 0.51 mm vs. 0.80 ± 0.28 mm), with statistical significance (*p* < 0.05) when contrasted with the healthy controls. Moreover, notable differences were observed in BMI, TC, TG, LDL‐C, aAIP, hsCRP, FIB, D‐dimer, and APTT between the two groups (*p* < 0.05).

**TABLE 1:** Comparison of demographic, anthropometric and laboratory data between patients with rheumatoid arthritis (RA) and healthy controls.

Parameters	Healthy controls (*n* = 105)	RA patients (*n* = 372)	*p*
Age (years)	58.06 ± 9.81	58.93 ± 11.99	0.496
Male sex, *n* (%)	24 (22.9%)	69 (18.5%)	0.325
Body mass index (kg/m^2^)	24.20 ± 2.25	24.95 ± 2.65	0.009
Systolic blood pressure (mm Hg)	128.10 ± 9.90	129.26 ± 18.25	0.532
Diastolic blood pressure (mm Hg)	78.08 ± 6.22	79.28 ± 12.09	0.329
Fasting blood glucose (mmol/L)	5.06 ± 0.66	5.16 ± 1.07	0.378
Total cholesterol (mmol/L)	4.67 ± 0.87	4.88 ± 0.98	0.036
Triglyceride (mmol/L)	1.42 ± 0.58	1.66 ± 1.00	0.019
HDL‐C (mmol/L)	1.55 ± 0.37	1.47 ± 0.41	0.067
LDL‐C (mmol/L)	2.38 ± 0.68	2.58 ± 0.88	0.028
aAIP	0.94 ± 0.24	1.02 ± 0.25	0.004
hsCRP (mg/L)	1.19 (0.51, 2.35)	8.14 (2.25, 31.13)	< 0.001
Prothrombin time (s)	11.47 ± 0.79	11.49 ± 0.95	0.895
APTT (s)	27.32 ± 2.32	26.70 ± 2.91	0.043
Thrombin time (s)	15.94 ± 1.10	15.55 ± 2.12	0.065
Fibrinogen (g/L)	2.64 (2.31, 3.01)	3.31 (2.63, 4.25)	< 0.001
D‐dimer (mg/L)	0.27 (0.20, 0.42)	0.83 (0.35, 1.98)	< 0.001
R time (min)	6.60 ± 0.95	6.51 ± 1.32	0.497
K time (min)	1.77 ± 0.41	1.70 ± 0.54	0.234
α angle (°)	63.36 ± 4.32	64.07 ± 7.59	0.360
MA (mm)	61.20 ± 4.45	62.31 ± 4.60	0.029
CIMT (mm)	0.80 ± 0.28	0.94 ± 0.51	0.009

*Note*: Data are given as mean ± SD, *n* (%), or median (IQR).

Abbreviations: aAIP, adjusted atherogenic index of plasma; APTT, activated partial thromboplastin time; CIMT, carotid intima‐media thickness; HDL‐C, high density lipoprotein cholesterol; hsCRP, high‐sensitive C‐reactive protein; K, kinetics; LDL‐C, low density lipoprotein cholesterol; MA, maximum amplitude; R, reaction.

### 3.2 Comparison of Characteristics Between RA Patients With Early AS and Those Without AS

The 372 patients with RA were divided into two cohorts based on CIMT measurements: an AS group of 270 patients with CIMT > 0.9 mm and a non‐AS group comprising 102 patients with CIMT ≤ 0.9 mm. The demographic, anthropometric, and laboratory characteristics of these groups were subsequently compared. As illustrated in Table 2, all parameters differed significantly in a statistical sense between the two groups (*p* < 0.05), except for PT, APTT, TT, and K time, which were non‐significant (*p* > 0.05). Regarding coagulation parameters, patients in the AS group demonstrated significantly elevated median levels of FIB (3.61 [2.77–4.86] g/L vs. 3.15 [2.60–3.94] g/L) and D‐dimer (1.09 [0.55–2.32] mg/L vs. 0.72 [0.30–1.81] mg/L), with greater mean values for the α angle (65.25° ± 7.22° vs. 63.14° ± 7.45°) and MA (64.90 ± 4.38 mm vs. 61.33 ± 4.30 mm), compared to the non‐AS group. A significantly shorter mean R time was observed in AS patients (6.24 ± 1.57 min vs. 6.61 ± 1.21 min).

**TABLE 2:** Comparison of demographic, anthropometric and laboratory data between rheumatoid arthritis patients with early atherosclerosis (AS) and those without AS.

Parameters	Non‐AS group (*n* = 270)	AS group (*n* = 102)	*p*
Age (years)	57.89 ± 12.13	61.67 ± 11.22	0.007
Male sex, *n* (%)	42 (15.6%)	27 (26.5%)	0.016
Body mass index (kg/m^2^)	24.49 ± 2.31	26.15 ± 3.08	< 0.001
Systolic blood pressure (mm Hg)	127.21 ± 16.49	134.69 ± 21.40	< 0.001
Diastolic blood pressure (mm Hg)	78.44 ± 11.27	81.48 ± 13.84	0.031
Fasting blood glucose (mmol/L)	5.07 ± 0.79	5.37 ± 1.56	0.018
Total cholesterol (mmol/L)	4.78 ± 0.97	5.17 ± 0.94	0.001
Triglyceride (mmol/L)	1.48 ± 0.60	2.14 ± 1.55	< 0.001
HDL‐C (mmol/L)	1.52 ± 0.40	1.32 ± 0.40	< 0.001
LDL‐C (mmol/L)	2.44 ± 0.78	2.95 ± 1.02	< 0.001
aAIP	0.97 ± 0.22	1.16 ± 0.29	< 0.001
hsCRP (mg/L)	6.98 (1.69, 27.42)	15.02 (2.96, 47.28)	0.005
Prothrombin time (s)	11.50 ± 0.99	11.44 ± 0.85	0.596
APTT (s)	26.78 ± 2.74	26.46 ± 3.32	0.341
Thrombin time (s)	15.59 ± 2.31	15.42 ± 1.51	0.473
Fibrinogen (g/L)	3.15 (2.60, 3.94)	3.61 (2.77, 4.86)	0.002
D‐dimer (mg/L)	0.72 (0.30, 1.81)	1.09 (0.55, 2.32)	0.001
R time (min)	6.61 ± 1.21	6.24 ± 1.57	0.018
K time (min)	1.77 ± 0.51	1.68 ± 0.60	0.168
α angle (°)	63.14 ± 7.45	65.25 ± 7.22	0.014
MA (mm)	61.33 ± 4.30	64.90 ± 4.38	< 0.001
CIMT (mm)	0.67 ± 0.12	1.63 ± 0.47	< 0.001

*Note*: Data are given as mean ± SD, *n* (%), or median (IQR).

Abbreviations: aAIP, adjusted atherogenic index of plasma; APTT, activated partial thromboplastin time; CIMT, carotid intima‐media thickness; HDL‐C, high density lipoprotein cholesterol; hsCRP, high‐sensitive C‐reactive protein; K, kinetics; LDL‐C, low density lipoprotein cholesterol; MA, maximum amplitude; R, reaction.

### 3.3 Multivariable Logistic Regression Analysis of Risk Factors for Early AS in Patients With RA

To identify the independent risk factors associated with early AS in RA patients, we performed a multivariate logistic regression analysis using CIMT > 0.9 as the dependent variable. Independent variables were selected based on their statistical significance in univariate analyses that compared AS and non‐AS groups. Variables exhibiting multicollinearity, indicated by a VIF ≥ 5, were excluded. The ultimate logistic regression model comprised these independent variables: age, sex, BMI, SBP, R time, α angle, MA, FIB, D‐dimer, hsCRP, FBG, TC, LDL‐C, and aAIP. Table 3 illustrates that BMI (odds ratio [OR], 1.167; 95% confidence interval [CI]: 1.033–1.317; *p* = 0.013), MA (OR, 1.219; 95% CI: 1.128–1.317; *p* < 0.001), FIB (OR, 1.426; 95% CI: 1.105–1.841; *p* = 0.006), LDL‐C (OR, 2.353; 95% CI: 1.370–4.040; *p* = 0.002), and a 0.1 unit increase in aAIP (OR, 1.490; 95% CI: 1.304–1.703; *p* < 0.001) were significantly associated with early AS in patients with RA.

**TABLE 3:** Multivariable logistic regression analysis of risk factors for early atherosclerosis in patients with rheumatoid arthritis.

	OR	95% CI	*p*	VIF
Age	1.019	0.991–1.048	0.190	1.187
Sex (male)	1.490	0.694–3.198	0.306	1.189
Body mass index	1.167	1.033–1.317	0.013	1.288
Systolic blood pressure	1.017	1.000–1.034	0.053	1.220
Fasting blood glucose	1.171	0.825–1.662	0.378	1.065
Total cholesterol	0.800	0.495–1.292	0.361	2.738
LDL‐C	2.353	1.370–4.040	0.002	2.723
aAIP (per 0.1 unit increase)	1.490	1.304–1.703	< 0.001	1.080
hsCRP	1.000	0.993–1.007	0.975	1.214
Fibrinogen	1.426	1.105–1.841	0.006	1.278
D‐dimer	1.029	0.985–1.076	0.201	1.112
R time	1.040	0.821–1.319	0.745	1.308
α angle	1.029	0.982–1.079	0.233	1.197
MA	1.219	1.128–1.317	< 0.001	1.075

Abbreviations: aAIP, adjusted atherogenic index of plasma; AS, atherosclerosis; CI, confidence interval; hsCRP, high‐sensitive C‐reactive protein; LDL‐C, low density lipoprotein cholesterol; MA, maximum amplitude; OR, odds ratio; R, reaction; VIF, variance inflation factor.

### 3.4 Diagnostic Efficacy of LDL‐C, aAIP, FIB, MA, and Their Combinations in Identifying Early AS Among RA Patients

**FIGURE 2:**
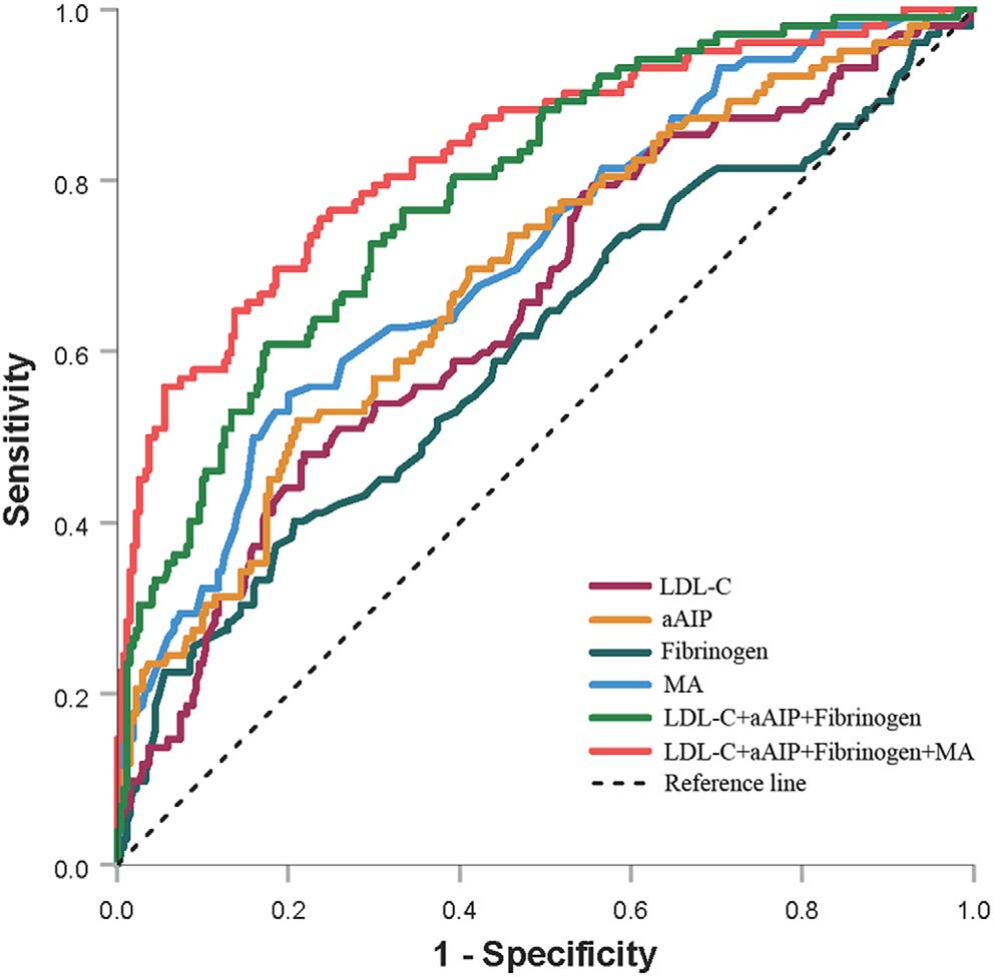
Diagnostic efficacy of LDL‐C, aAIP, FIB, MA, and their combinations in identifying early atherosclerosis among patients with rheumatoid arthritis. aAIP, adjusted atherogenic index of plasma; LDL‐C, low density lipoprotein cholesterol; MA, maximum amplitude.

The diagnostic performance of independent risk factors in distinguishing AS from non‐AS conditions among RA patients was performed using ROC analysis. The study assessed the efficacy of biological markers—including MA, FIB, LDL‐C, and aAIP—in two combined biomarker panels: a three‐marker panel (MA, FIB, LDL‐C) and a four‐marker panel (MA, FIB, LDL‐C, aAIP). ROC analysis revealed AUC values of 0.711, 0.605, 0.653, 0.689, 0.787, and 0.831 for MA, FIB, LDL‐C, aAIP, the three‐marker combination, and the four‐marker combination, respectively, in detecting AS. Optimal cut‐off values for MA, FIB, LDL‐C, and aAIP were determined as 62.85, 4.20, 2.99, and 0.99, yielding sensitivities of 71.5%, 60.2%, 68.1%, and 70.3%, and specificities of 63.5%, 59.6%, 58.7%, and 69.8%, respectively. The three‐marker panel exhibited 74.8% sensitivity and 71.4% specificity, while the four‐marker panel displayed 77.6% sensitivity and 75.3% specificity. These results are summarized in Table 4 and Figure 2.

**TABLE 4:** Comparison of receiver operating characteristic analysis of LDL‐C, aAIP, Fibrinogen, MA, and their combinations for detecting early atherosclerosis in patients with rheumatoid arthritis.

Variables	AUC (95% CI)	*p*	Cut‐off values	Sensitivity	Specificity
LDL‐C	0.653 (0.590–0.716)	< 0.001	2.99	68.1%	58.7%
aAIP	0.689 (0.628–0.750)	< 0.001	0.99	70.3%	69.8%
Fibrinogen	0.605 (0.538–0.673)	0.002	4.20	60.2%	59.6%
MA	0.711 (0.652–0771)	< 0.001	62.85	71.5%	63.5%
LDL‐C + aAIP + Fibrinogen	0.787 (0.737–0.838)	< 0.001	/	74.8%	71.4%
LDL‐C + aAIP + Fibrinogen + MA	0.831 (0.782–0.880)	< 0.001	/	77.6%	75.3%

Abbreviations: aAIP, adjusted atherogenic index of plasma; AUC, the area under the curve; CI, confidence interval; LDL‐C, low‐density lipoprotein cholesterol; MA, maximum amplitude.

## 4 Discussion

The research provided the first detailed examination of the association between TEG parameters and AS in individuals suffering from RA, while innovatively incorporating the aAIP into this research area. Our findings indicate that among multiple biological markers demonstrating independent positive associations with AS in patients with RA, including MA, aAIP, LDL‐C, and FIB, MA exhibited superior discriminative capacity for disease detection. Notably, the combined diagnostic model that integrated MA with the other three parameters exhibited significantly enhanced predictive performance compared to individual biomarkers or combinations that excluded MA. These results establish MA as a pivotal biomarker for AS in patients with RA while highlighting the clinical significance of multimodal biomarker integration for optimized diagnostic accuracy in this high‐risk population.

With the exception of MA, TEG parameters were similar between patients with RA and healthy controls. In contrast, patients with RA exhibited shortened APTT along with elevated D‐dimer and FIB levels among the conventional coagulation parameters. This comparison suggests that TEG does not offer a distinct advantage in assessing hypercoagulability in patients with RA, and these coagulation parameters lack equivalent clinical significance in this evaluation. Multiple studies have demonstrated that patients with hypercoagulable states may display diverse alterations in coagulation parameters, as seen in TEG and conventional coagulation tests. Jiaao et al. [19] reported that patients with pancreatic cancer often exhibit marked hypercoagulability, characterized by significantly elevated D‐dimer, FIB, MA, and α‐angle levels, along with shortened APTT, R time, and K time. A recent study reported that patients with gestational diabetes mellitus exhibited hypercoagulability, as indicated by APTT, FIB, and lysis at 30 min (LY30)—a different TEG parameter—but not MA or other TEG parameters [20]. Despite numerous TEG parameters documented in the literature concerning their effectiveness in assessing hypercoagulability or the risk of venous thromboembolism (VTE), two recent systematic reviews have determined that MA is a more reliable indicator of these adverse events than other TEG parameters, including R time, K time, and α‐angle [21, 22].

This study demonstrates a significant increase in MA, α angle, FIB, and D‐dimer levels, along with a marked reduction in R time, in the AS group versus the non‐AS group among patients with RA. These findings indicate that MA and α angle in TEG parameters and FIB and D‐dimer in conventional coagulation tests can be biomarkers for predicting AS in patients with RA. However, subsequent logistic regression analysis revealed that only MA and FIB exhibited a significant and positive correlation with the risk of early AS among the specified coagulation parameters. This indicates that MA and FIB are independent of other coagulation factors and are predictors of early‐stage AS. While FIB was somewhat less effective than MA in detecting early AS in this study, its role in AS development is significant. FIB increases blood viscosity, stimulates fibrin synthesis, and enhances interactions between platelets and lipids, contributing to AS onset and progression [23]. A recent cross‐sectional study identified FIB as an independent marker of early AS in overweight individuals with dyslipidemia [24]. This study is the first report of MA as an indicator of early AS; previous research has highlighted its efficacy in predicting other disease progression or prognosis. A research conducted by Zhang and Zhuang [25] revealed that MA had a high predictive value for recurrent spontaneous abortion, with an AUC of 0.833. When the MA cutoff was set at 63.0, it achieved a sensitivity of 70.4% and a specificity of 86.4%. Zhong et al. [26] introduced a new parameter, “time to maximum amplitude (TMA),” which measures the time needed to reach MA. Unlike TEG and traditional coagulation parameters, TMA alone predicted mortality risk of death in COVID‐19 patients. As previously mentioned, MA is primarily influenced by platelets [12, 13]. The strong correlation between MA and the risk of early AS may be attributed to its close association with platelet activation and function. There is compelling evidence implicating the role of platelets in AS progression. During the initiation of AS, platelets adhere to damaged endothelial cells, serving as an intermediary between leukocytes and these cells [27]. Platelets have been demonstrated to stimulate the transformation of monocytes into activated macrophages and promote the formation of foam cells, which are critical in all stages of AS development and significantly contribute to plaque formation [28]. A previous study indicates that platelets can migrate independently to sites of vascular injury and inflammation, where they are recruited to atherosclerotic plaques [29].

Our study demonstrated that aAIP constitutes an independent risk factor for early AS. Recently, AIP/aAIP has gained widespread recognition as potent predictors of AS in both healthy and diseased populations [30, 31]. Nonetheless, our findings indicated that, compared to MA, aAIP did not exhibit a noteworthy advantage in the early detection of AS among patients with RA. Presently, the exploration of the connection between AIP/aAIP and AS, specifically among those with RA, is still in its infancy. Nevertheless, AIP has already established itself as an independent marker of early AS in other autoimmune disorders, such as systemic lupus erythematosus (SLE) [32]. Furthermore, our study confirmed that BIM and LDL‐C are independent risk factors that contribute to increased CIMT, which is in agreement with prior research findings [33].

Our study is subject to several limitations. First, the cross‐sectional design precluded the longitudinal assessment of changes in coagulation parameters about disease severity and hindered causal inferences. Second, while patients receiving steroids and immunosuppressants were excluded, the potential impact of other medications or therapeutic interventions on the study outcomes cannot be entirely ruled out. Third, as a single‐center retrospective study, selection bias is possible.

## 5 Conclusion

This study demonstrates that hypercoagulability significantly influences AS progression and identifies MA as a novel and effective marker for the early detection of AS in patients with RA. Furthermore, the simultaneous evaluation of MA, FIB, LDL‐C, and aAIP can improve the diagnostic accuracy for early AS by enhancing sensitivity and specificity. Future multicenter or prospective studies should investigate the influence of clinical interventions on coagulation parameters.

## Conflicts of Interest

The authors declare no conflicts of interest.

## Data Availability

The data that support the findings of this study are available from the corresponding author upon reasonable request.
